# TMI-1, TNF-α-Converting Enzyme Inhibitor, Protects Against Paclitaxel-Induced Neurotoxicity in the DRG Neuronal Cells *In Vitro*

**DOI:** 10.3389/fphar.2022.842779

**Published:** 2022-02-16

**Authors:** Yesul Kim, Young-Hoon Jung, Seung-Bin Park, Heekee Kim, Jae-young Kwon, Hae-kyu Kim, Hyeon-Jeong Lee, Soeun Jeon, Eunsoo Kim

**Affiliations:** ^1^ Department of Anesthesia and Pain Medicine, School of Medicine, Pusan National University, Busan, South Korea; ^2^ Department of Anesthesia and Pain Medicine, Biomedical Research Institute, Pusan National University Hospital, Busan, South Korea; ^3^ Department of Pain Medicine, The University of Texas MD Anderson Cancer Center, Houston, United States

**Keywords:** CIPN, paclitaxel, dorsal root ganglion, 50B11, TNF-α, TRPV1, neuroinflammation, TMI-1

## Abstract

**Background:** Chemotherapy-induced peripheral neuropathy (CIPN) negatively impacts cancer survivors’ quality of life and is challenging to treat with existing drugs for neuropathic pain. TNF-α is known to potentiate TRPV1 activity, which contributes to CIPN. Here, we assessed the role of TMI-1, a TNF-α-converting enzyme inhibitor, in paclitaxel (PAC)-induced neurotoxicity in dorsal root ganglion (DRG) cells.

**Materials and Methods:** Immortalized DRG neuronal 50B11 cells were cultured and treated with PAC or PAC with TMI-1 following neuronal differentiation. Cell viability, analysis of neurite growth, immunofluorescence, calcium flow cytometry, western blotting, quantitative RT-PCR, and cytokine quantitation by ELISA were performed to determine the role of TMI-1 in neurotoxicity in neuronal cells.

**Results:** PAC administration decreased the length of neurites and upregulated the expression of TRPV1 in 50B11 cells. TMI-1 administration showed a protective effect by suppressing inflammatory signaling, and secretion of TNF-α.

**Conclusion:** TMI-1 partially protects against paclitaxel-induced neurotoxicity by reversing the upregulation of TRPV1 and decreasing levels of inflammatory cytokines, including TNF-α, IL-1β, and IL-6 in neuronal cells.

## Introduction

Paclitaxel (PAC) is one of the most widely used chemotherapeutic agents against several types of cancers, including ovarian, breast, esophageal, lung, and other solid cancers (Mekhail and Markman, 2002). However, it commonly causes chemotherapy-induced peripheral neuropathy (CIPN), which presents as a typical glove and stocking neuropathy. The prevalence of CIPN is as high as 68% within the first month, and approximately 30% of patients will have persistent problems for more than 6 months after chemotherapy (Seretny et al., 2014). In addition, CIPN could affect the treatment of patients with cancer due to the limitation of dose, chemotherapeutic agent selection, or even discontinuation of treatment. Unfortunately, until recently, there have been no definite drugs available to treat or prevent CIPN, even though a well-designed clinical study reported that duloxetine has shown a significant reduction in pain (Smith et al., 2013).

The underlying mechanism of paclitaxel-induced peripheral neuropathy (PIPN) is not fully understood, but neuroinflammation and increased ion channel activity have been well identified as possible mechanisms in previous studies (Sisignano et al., 2014). PAC increases the levels of inflammatory cytokines, including tumor necrosis factor α (TNF-α) and interleukin-1β (IL-1β), in the lumbar dorsal root ganglion (DRG) of rats (Kim et al., 2019). Furthermore, PAC treatment also induces upregulation of transient receptor potential cation channel subfamily V member 1 (TRPV1) expression in DRG neurons, contributing to PIPN (Hara et al., 2013). In addition, TNF-α potentiates TRPV1 activity by sensitizing it (Rozas et al., 2016; Wang et al., 2018). Thus, we hypothesized that inhibition of TNF-α secretion alleviates paclitaxel-induced neurotoxicity by attenuating TRPV1 overexpression.

TNF-α is synthesized as a transmembrane protein and released into the soluble form by proteolytic cleavage of a disintegrin and metalloprotease 17 (ADAM17), called the TNF-α-converting enzyme (TACE). TMI-1 is a TACE inhibitor that prevents the secretion of soluble TNF-α. It shows tumor-selective cytotoxic action against breast cancer and other neoplasms (Mezil et al., 2012). It also demonstrated the potential to treat rheumatoid arthritis by showing that TMI-1 effectively reduces clinical severity scores in a mouse model of collagen-induced arthritis when used as a preventive and therapeutic strategy (Zhang et al., 2004). However, the therapeutic effect of TMI-1 on CIPN has not yet been investigated.

In the present study, we evaluated the protective effect of TMI-1 on paclitaxel-induced neurotoxicity in differentiated 50B11 cells, which are immortalized DRG neuronal cell lines derived from rats on embryonic day 14.5 (Chen et al., 2007). In addition, we investigated the underlying mechanism or association between TMI-1 and TRPV1 expression in DRG sensory neuronal cells.

## Materials and Methods

### Cell Culture and Drug Treatment

The 50B11 cells, a friendly gift from Dr. Ahmet Höke, were used as a model of DRG neurons and cultured in neurobasal medium (Life Technologies, Gibco, CA, United States) supplemented with 10% FBS (Hyclone, Cytiva, MA, United States), 2% B27 supplement (Life Technologies, Gibco, CA, United States), 11 mM d-glucose (Life Technologies, Gibco, CA, United States), 0.2% l-glutamine, and 1% antimycotic antibiotics (Life Technologies, Gibco, CA, United States). The cells were plated for 24 h before differentiation and experimentation, and the exhausted medium was replaced by a serum-free medium. For neuronal growth of the 50B11 cells, 75 μM forskolin (FSK, Abcam, Cambridge, United Kingdom) was used, which was dissolved in DMSO: ethanol = 1: 1 solution. Cells were treated with PAC (GenDEPOT, Katy, TX, United States) or PAC with TMI-1 (Sigma-Aldrich, St. Louis, MO, United States). The final concentrations of PAC and TMI-1 were 1, 10, and 100 ng/ml and 0.04, 0.4, and 4 ng/ml (0.1, 1, and 10 nM), respectively.

### Cell Viability Assay

Cell viability was determined using the EZ-CYTOX cell viability, proliferation, and cytotoxicity assay kit (DoGenBio, Seoul, Korea) according to the manufacturer’s instructions. Rat-immortalized DRG neuronal cells were cultured and seeded at 4 × 10^4^ cells in a 96-well plate. The cells stabilized and adhered to the wall, the serum-free media was changed, and the cells were treated with PAC (0–100,000 ng/ml) and TMI-1 (0–40 ng/ml) for 24, 48, and 72 h. The assay was performed in triplicate, and the results were expressed as absorbance at 450 nm. The fold-number of living cells could be determined by comparing these results with those of the untreated control.

### Analysis of Neurite Growth

Frontiers cells were seeded in 24-well plates to measure neurite growth for 24 h and imaged using a Nikon E300 microscope. The wells were photographed in five randomly selected fields at ×100 magnification for analysis. Images were analyzed using the ImageJ software.

### Immunofluorescence

Cells were seeded on coverslips in six-well plates coated with collagen and allowed to attach. After attachment, the cells were washed with washing buffer (PBS with 0.1% Tween 20), fixed with 2% paraformaldehyde, permeabilized with 0.01% Triton X-100, and blocked with blocking buffer (PBS with 10% FBS) at room temperature. The coverslips were incubated with primary antibodies [TRPV1 (Abclonal, MA, United States) and βIII tubulin (Abclonal, MA, United States)] overnight at 4°C, washed three times, and incubated with secondary antibodies [Alexa 488 (Abcam, Cambridge, United Kingdom) and Alexa 647 (Abcam, Cambridge, United Kingdom)] at room temperature. Cells were mounted in Prolong Gold antifade reagent with DAPI (Invitrogen, CA, United States) and imaged using a laser-scanning microscope (Leica Sp8 confocal microscope, Germany).

### Ca^2+^ Flow Cytometry and Ca^2+^ Imaging

For flow cytometry, the cells were incubated for 24 h, differentiated with FSK, and treated with PAC and PAC with TMI-1. Intracellular calcium levels were detected using the EZCell Calcium Detection Kit (Biovision, CA, United States) following the manufacturer’s protocol. The cells were resuspended in PBS and the fluorescence intensity of the calcium indicator in cells was measured by flow cytometry (BD FACSCanto, BD Biosciences); a minimum of 10,000 events were counted for each sample. For calcium imaging, cells were seeded in six-well plates, loaded with calcium indicators (EZCell Calcium Detection Kit), and washed with PBS. The stained cells were observed under a confocal microscope (green).

### Western Blotting

For whole protein quantification, the control and treated cells were scraped off the plates in the presence of ice-cold lysis buffer (PRO-PREP, Intron, Gyeonggi-do, Korea) with phosphatase inhibitor cocktail III (Calbiochem, United States). For nuclear protein quantification, the cells were lysed in hypotonic buffer (10 mM HEPES/KOH, 2 mM MgCl_2_, 0.1 mM EDTA, 10 mM KCl, and protease inhibitor), incubated on ice, and centrifuged, and the cytosolic protein was then separated. Pellets were washed entirely with PBS, suspended in ice-cold saline buffer (50 mM HEPES/KOH, 50 mM KCl, 300 mM NaCl, 0.1% glycerol, and protein inhibitor), incubated on ice, sonicated, and centrifuged, and used to separate the nuclear protein.

The protein lysates were separated by SDS-PAGE and electroblotted onto a nitrocellulose membrane (GeneDepot, United States). Blots were incubated with the corresponding primary antibodies (TRPV1, PKC, PI3K, NF-κB: Abclonal; pAKT, pERK, pJNK, pp38: cell Signaling) and subsequently with the secondary antibody [HRP-goat anti-rabbit IgG (Abclonal)]. Immunoreactive bands were visualized using an enhanced chemiluminescence peroxidase substrate solution (TransLab, Korea). The relative densities of the bands were normalized and quantified with β-actin using the ImageJ software.

### Isolation of Total RNA and Quantitative RT-PCR

Total RNA was isolated using TRIzol reagent (Invitrogen, CA, United States) according to the manufacturer’s protocol. The concentration of total RNA was determined using a Nanodrop spectrophotometer (BioTek Instruments Inc., VT, United States). RNA was reverse transcribed into cDNA using Maxime RT PreMix (Intron, Gyeonggi-Do, Korea). Quantitative PCR was performed using Brilliant III SYBR Green QPCR kit (Agilent, CA, United States) on an Applied Biosystems Real-Time PCR system (Life Technologies, CA, United States). The Ct values were determined for each product and normalized against the Ct value of the reference gene (GAPDH). The values were calculated using ΔΔCT, where Ct is the cycle threshold. The primer sequences were as follows: IL-1β sense CAC​CTC​TCA​AGC​AGA​GCA​CAG, antisense GGG​TTC​CAT​GGT​GAA​GTC​AAC; IL-6 sense TCC​TAC​CCC​AAC​TTC​CAA​TGC​TC, antisense TTG​GAT​GGT​CTT​GGT​CCT​TAG​CC; TNF-α sense AAA​TGG​GCT​CCC​TCT​CAT​CAG​TTC, antisense TCT​GCT​TGG​TGG​TTT​GCT​ACG AC; GAPDH sense GTA​TTG​GGC​GCC​TGG​TCA​CC, and antisense CGC​TCC​TGG​AAG​ATG​GTG​ATG​G.

### Cytokine Quantitation by ELISA

To quantify TNF-α secretion, sandwich ELISA was performed according to the manufacturer’s instructions. First, TNF-α was measured in the culture medium using the Rat TNF-α Pre-coated ELISA Kit (Biogems, CA, United States). Next, cells were analyzed using a 450 nm ELISA microplate reader (BioTek Instruments Inc., VT, United States). The cells were quantified in the culture medium and a standard curve was used.

### Statistical Analysis

All analyses were performed using MedCalc^®^ Statistical Software for Windows, version 18.11.6 (MedCalc Software Ltd., Ostend, Belgium). After testing for normal distribution, data were presented as the median and interquartile range (IQR) and analyzed using the Mann-Whitney *U* test or Kruskal-Wallis test. If the differences between the groups were significant, the Conover tests were performed for post hoc multiple comparisons. Finally, the Jonckheere-Terpstra trend test was performed to demonstrate the dose-response relationship [i.e. (Mekhail and Markman, 2002) from FSK to P100 (Seretny et al., 2014); from P100 to P100 + T4]. Two-sided *p*-values < 0.05 were considered to indicate statistical significance.

## Results

### Effects of Paclitaxel and TMI-1 on the Viability of 50B11 Cells

Cell viability assays were performed to determine the optimal concentrations of paclitaxel and TMI-1. Varying concentrations of paclitaxel (0–100,000 ng/ml) and TMI-1 (0–40 ng/ml) were added to the rat-immortalized DRG neuronal cell lines and incubated for 24, 48, and 72 h. Paclitaxel at concentrations up to 100 ng/ml did not affect cell viability (data not shown). TMI-1 at concentrations up to 4 ng/ml was ineffective in modulating cell viability except for at 72 h (data not shown). The final concentrations of paclitaxel that cause neurotoxicity but do not affect cell viability were determined to be 1, 10, and 100 ng/ml. In addition, the concentrations of TMI-1 were optimized to 0.04, 0.4, and 4 ng/ml.

### Effects of TMI-1 on the Neurite Growth of 50B11 Cells Treated With Paclitaxel

**FIGURE 1:**
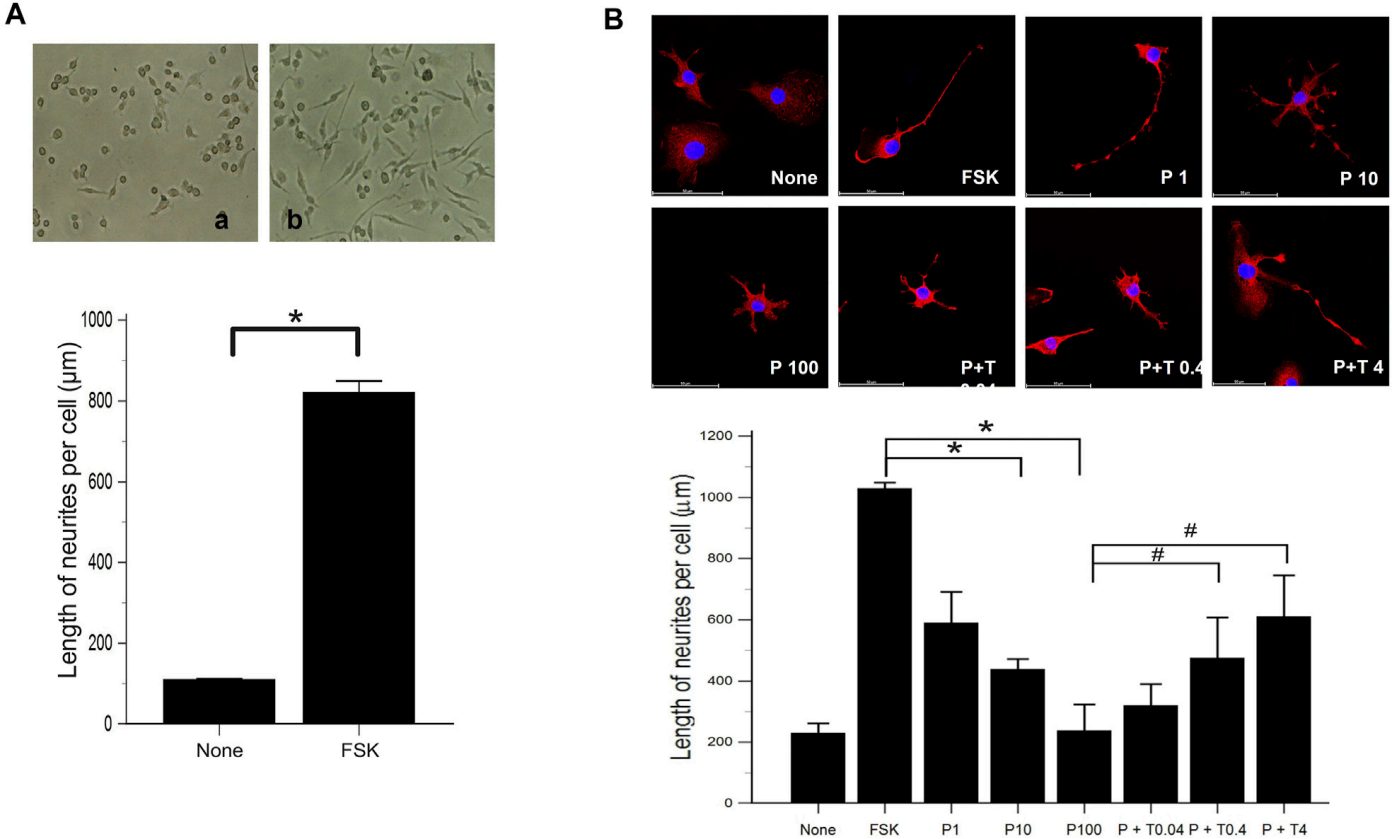
Morphological differentiation of 50B11 cells **(A)** Low magnification images illustrate the high efficiency of morphological differentiation using FSK. Micrographs showing 50B11 cells cultured without mitogenic agents (no treatment) and the presence of 75 µM FSK (*n* = 5 per group). **(B)** By immunofluorescence, the cells were stained for βIII-tubulin (red), and the cell nuclei were counterstained with DAPI (blue). Cells were cultured in the absence of mitogenic agents and the presence of 75 µM forskolin. Differentiated cells were treated with 1, 10, and 100 ng/ml PAC and 100 ng/ml PAC with 0.04, 0.4, and 4 ng/ml TMI-1 (Scale bar = 50 µm). Analysis of neurite length in 50B11 cells showed differences in undifferentiated cells’ morphological characteristics compared to differentiated cells. Data were presented as the median and interquartile range (IQR) and analyzed using the Mann-Whitney *U* test or Kruskal-Wallis test followed by the Conover test (*n* = 4 per group). *p < 0.05 compared to the FSK; ^#^ *p* < 0.05 compared to the P100.

We evaluated the effect of forskolin on the morphological differentiation of 50B11 cells using low magnification images. Compared to 50B11 cells cultured without any mitogenic agents, the cells treated with 75 µM forskolin showed increased neurite length per cell (Figure 1A; *p* = 0.0090 by Mann-Whitney *U* test).

Morphological changes were observed 24 h after treatment of 50B11 cells with paclitaxel (100 ng/ml) and paclitaxel with TMI-1 (4 ng/ml). In the immunofluorescence analysis, the cells treated with paclitaxel showed degeneration as blebbing of axons, and some recovery of axonal injury was observed after administration of TMI-1 (Figure 1B). Compared to non-treated cells, extended lengths of neurites were observed in 50B11 cells treated with forskolin. Treatment of paclitaxel reduced the length of neurites compared to that by treatment with forskolin, and neurite growth improved after administration of TMI-1. In addition, both paclitaxel and TMI-1 showed a dose-dependent effect on neurite growth (Figure 1B; *p* = 0.001618 by Kruskal–Wallis test From FSK to P100, *p* = 0.00004, From P100 to P100 + T4, *p* = 0.002 by Jonckheere-Terpstra trend test).

### Effects of Paclitaxel and TMI-1 on TRPV1 Expression of 50B11 Cells

**FIGURE 2:**
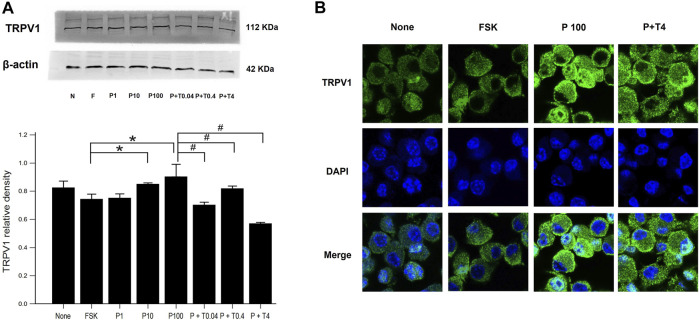
TRPV1 expression in 50B11 cells. **(A)** Western blot analysis for TRPV1. Quantification of the basal levels of TRPV1 relative to those of β-actin. **(B)** TRPV1 (green) expression in a non-treated 50B11 cell; only FSK, PAC (100 ng/ml) on differentiated cells, and PAC (100 ng/ml) with TMI-1 (4 ng/ml) on differentiated cells. Cells were stained with Alexa Fluor 488, and the nucleus was counterstained with DAPI (blue). Data were presented as the median and interquartile range (IQR) and analyzed using the Kruskal-Wallis test followed by the Conover test (*n* = 3 per group). *p < 0.05 compared to the FSK; ^#^ *p* < 0.05 compared to the P100.

The estimation of TRPV1 expression is shown in Figure 2. Western blot analysis (Figure 2A) revealed that the protein level of TRPV1 was increased in the cells treated with 10 and 100 ng/ml paclitaxel in a dose-dependent manner compared with that in the cells treated with only 75 µM forskolin. All cells treated with 100 ng/ml paclitaxel with different doses of TMI-1 (0.04, 0.4, 4 ng/ml) showed a significant decrease in TRPV1 protein level, which showed a dose-dependent trend, compared with that in the cells treated with 100 ng/ml paclitaxel (Figure 2A; *p* = 0.005372 by Kruskal–Wallis test; From FSK to P100, *p* = 0.000108, From P100 to P100 + T4, *p* = 0.01052 by Jonckheere-Terpstra trend test). In immunofluorescence assays (Figure 2B), the expression of TRPV1 was observed predominantly in the membrane of 50B11 cells treated with 100 ng/ml paclitaxel. The expression of TRPV1 was reversed by the administration of 4 ng/ml TMI-1 compared with administration of paclitaxel alone.

### Effects of Paclitaxel and TMI-1 on Calcium Influx of 50B11 Cells

**FIGURE 3:**
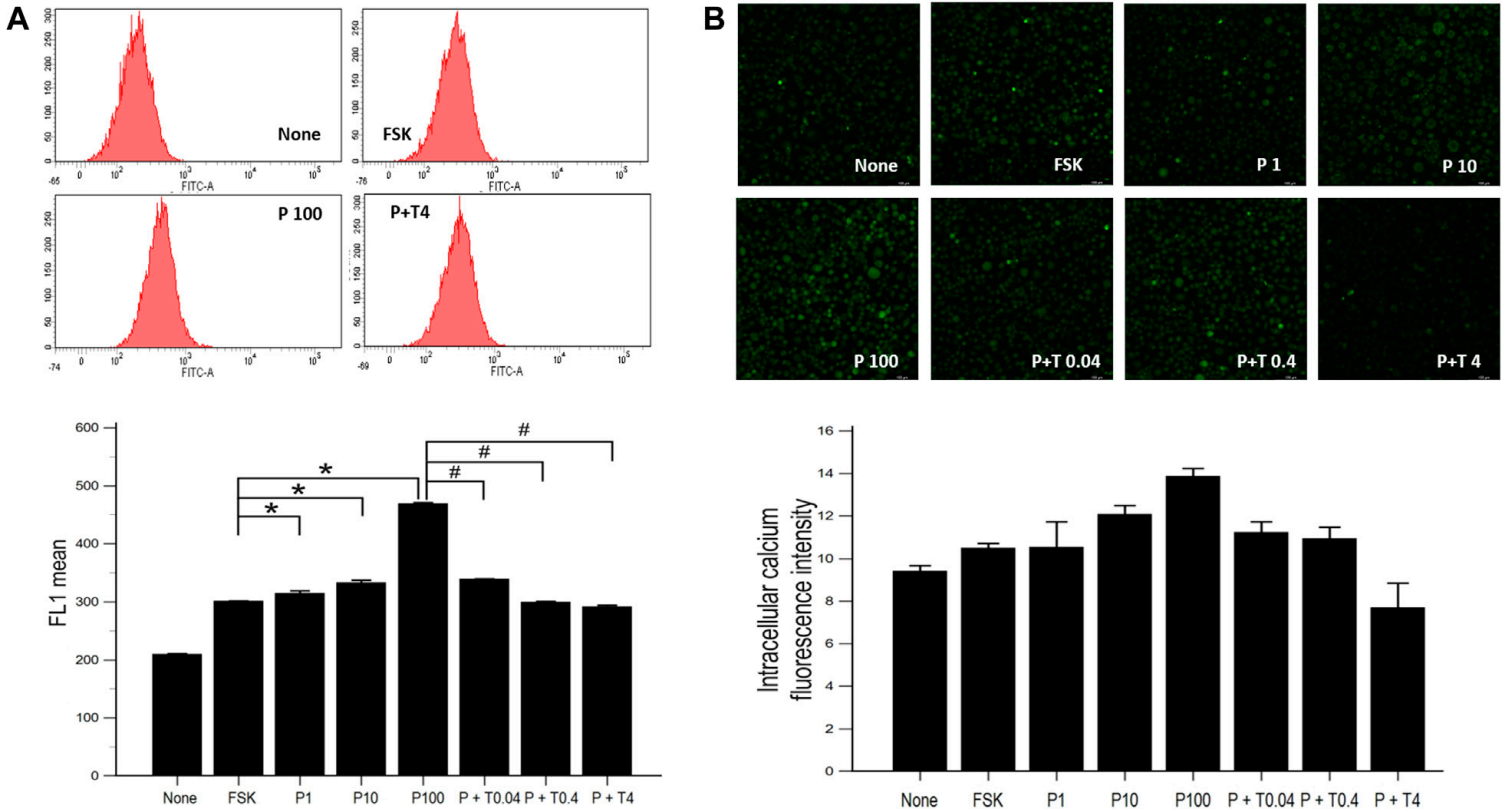
Calcium influx in 50B11 cells. Intracellular calcium levels were evaluated in FSK-derived neurons under serum-free conditions. **(A)** Histograms from flow cytometry analysis. The stained cell population and mean fluorescence intensity in FL1 were quantified and compared among undifferentiated cells, differentiated cells, PAC treated differentiated cells, and PAC with TMI-1 treated differentiated cells (*n* = 3 per group). **(B)** Calcium-stained cells were green under the confocal microscope (*n* = 2 per group). Data are presented as the median and interquartile range (IQR) and analyzed using the Kruskal-Wallis test followed by the Conover test. *p < 0.05 compared to the FSK; ^#^ *p* < 0.05 compared to the P100.

The increased concentration of intracellular calcium level was shown by flow cytometry in cells treated with 1, 10, 100 ng/ml paclitaxel, showing a dose-dependent trend. After treatment with TMI-1 (0.04, 0.4, and 4 ng/ml), the concentration of intracellular calcium decreased in a dose-dependent manner (Figure 3A; *p* = 0.002338 by Kruskal–Wallis test; From FSK to P100, *p* = 0.00012, From P100 to P100 + T4, *p* = 0.00012 by Jonckheere-Terpstra trend test). Fluorescence microscopy images showed that intracellular calcium increased in cells treated with paclitaxel and decreased in those treated with TMI-1 with a dose-dependent trend. However, the quantified intracellular calcium fluorescence intensity was not statistically significant (Figure 3B; *p* = 0.093320 by Kruskal-Wallis test; From FSK to P100, *p* = 0.02154, From P100 to P100 + T4, *p* = 0.00012 by Jonckheere-Terpstra trend test).

### Effects of Paclitaxel and TMI-1 on Neuroinflammatory Signaling Pathway of 50B11 Cells

**FIGURE 4:**
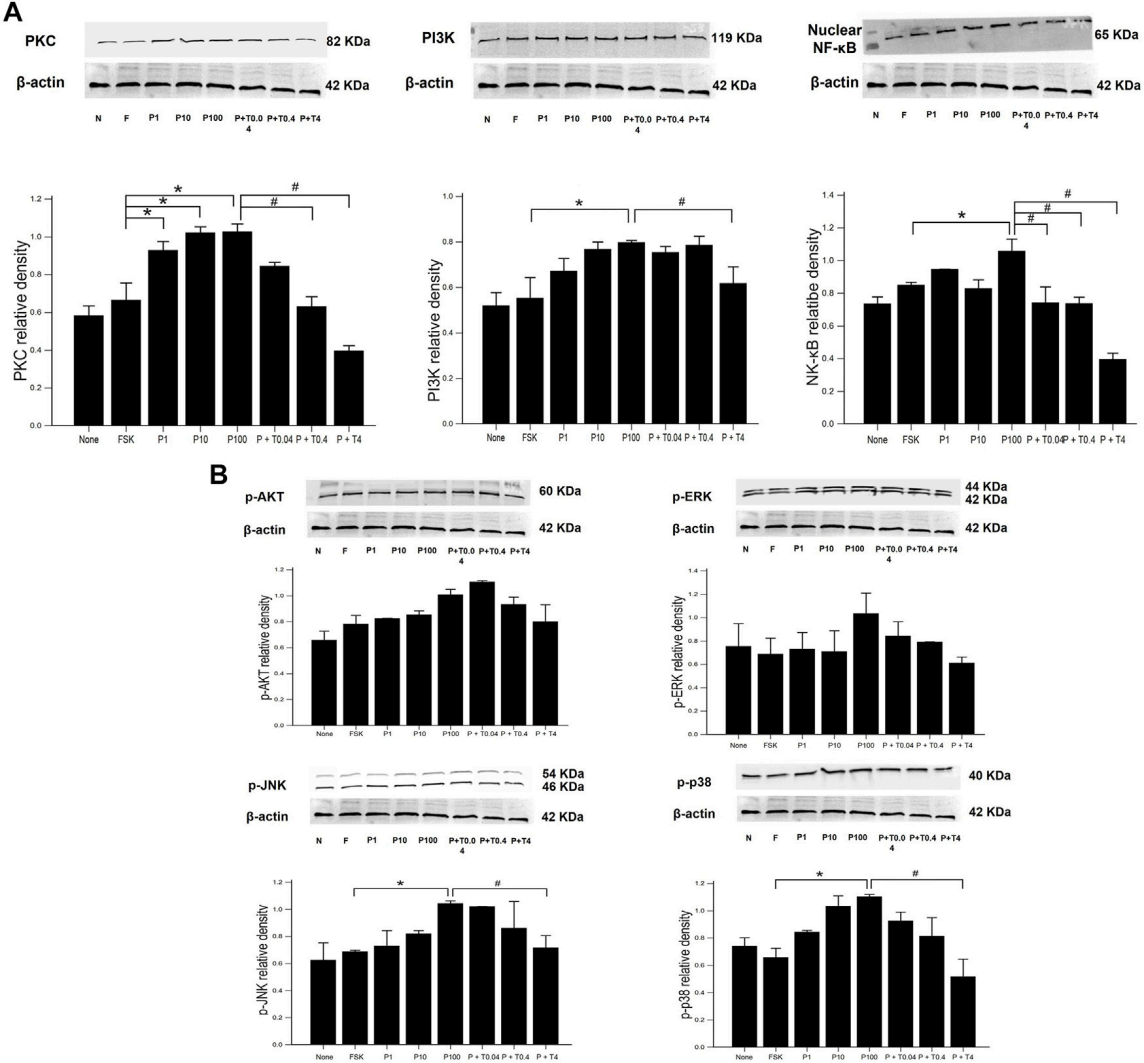
Western blot analysis of neuronal inflammatory signal on 50B11 cells. Western blot images showing the relative protein expression of signaling protein, including **(A)** PKC, PI3K, NF-κB, and **(B)** phospho-MAPK (ERK, JNK, and p38) relative to that of β-actin. ImageJ-quantified densitometry. Data were presented as the median and interquartile range (IQR) and analyzed using the Kruskal-Wallis test followed by the Conover test (*n* = 3 per group). *p < 0.05 compared to the FSK; ^#^ *p* < 0.05 compared to the P100.

The measurement of signaling protein levels, including PKC, PI3K, nuclear NF-κB, and phospho-MAPK (ERK, JNK, and p38), is shown in Figure 4. Compared with that of forskolin, the levels of PKC was significantly increased in the cells treated with 1, 10, and 100 ng/ml paclitaxel (*p* < 0.05) (Figure 4A). The protein levels of NF-κB were not significantly increased in the cells treated with 1 and 10 ng/ml paclitaxel, though they were when the cells were treated with 100 ng/ml paclitaxel compared to those in cells treated with forskolin (*p* < 0.05). Treatment with 4 ng/ml TMI-1 decreased the levels of PKC, PI3K, and NF-κB (*p* < 0.005, *p* < 0.05, and *p* < 0.005, respectively). The low dose (0.04 ng/ml) of TMI-1 did not show a statistically significant decrease in all phospho-MAPK levels (Figure 4B). Neither paclitaxel nor TMI-1 contributed to the expression of *p*-AKT. The protein levels of *p*-ERK, *p*-JNK, and p-p38 showed a significant increase after 100 ng/ml paclitaxel treatment (*p* < 0.05) and a significant decrease after treatment with 4 ng/ml TMI-1 (*p* < 0.05).

**FIGURE 5:**
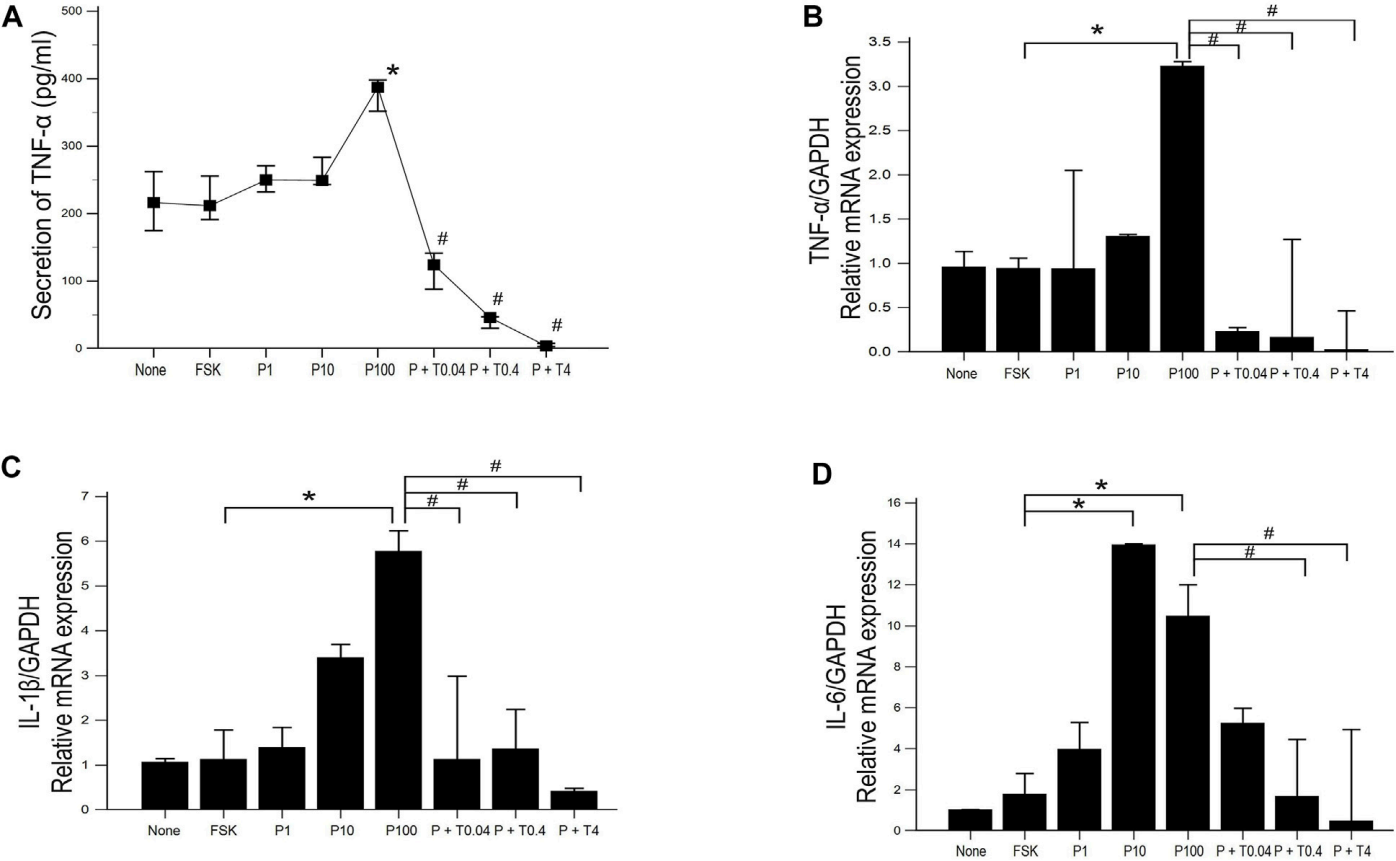
Expression and secretion of proinflammatory cytokine in 50B11 cells. **(A)** TNF-α secretion was determined by ELISA in culture medium under serum-free conditions and different treatments (*n* = 3 per group; ^#^ *p* < 0.05 compared to the P100). **(B–D)** Total RNA was isolated, and quantitative PCR was performed. Ct (threshold cycle) values were calculated, and the ∆Ct method was used for relative mRNA quantification. The expression levels of genes were normalized to that of GAPDH and showed a significant increase in the mRNA levels of the proinflammatory cytokines tumor necrosis factor (TNF)-α, interleukin (IL)-1β, and IL-6 (*n* = 3 per each group). Data were presented as the median and interquartile range (IQR) and analyzed using the Kruskal-Wallis test followed by the Conover test. *p < 0.05 compared to the FSK; ^#^ *p* < 0.05 compared to the P100.

ELISA was used to estimate the level of TNF-α secretion under different treatment conditions. Secretion of TNF-α was significantly increased in 50B11 cells treated with 100 ng/ml paclitaxel and decreased in the cells treated with 0.04, 0.4, and 4 ng/ml TMI-1, showing dose-dependent trend (Figure 5A; *p* = 0.004502 by Kruskal–Wallis test; From FSK to P100, *p* = 0.00692, From P100 to P100 + T4, *p* = 0.00012 by Jonckheere-Terpstra trend test). The expression of proinflammatory cytokines, including TNF-α, IL-1β, and IL-6, was measured by quantitative PCR (Figures 5B–D). 50B11 cells treated with 100 ng/ml paclitaxel showed a significant increase in the mRNA levels of those cytokines compared to forskolin. In contrast, treatment with TMI-1 (0.4, 4 ng/ml) caused a significant decrease in the expression of proinflammatory cytokines compared to that after treatment with 100 ng/ml paclitaxel (Figures 5B–D; *p* = 0.021703, *p* = 0.041483, *p* = 0.023810 by Kruskal-Wallis test).

## Discussion

The present study was performed to demonstrate the protective effect of TMI-1 against paclitaxel-induced neurotoxicity. Paclitaxel induced axonal degeneration, which was identified by axonal blebbing and shortening of the length of neurites, and TMI-1 attenuated these changes in DRG neuronal cells. In line with previous research results that demonstrate that paclitaxel induces an increase in TRPV1 expression (Hara et al., 2013; Li et al., 2015a; Kalynovska et al., 2017), the increase in expression of TRPV1 *via* paclitaxel-induced neurotoxicity was confirmed through western blot analysis and immunohistochemistry. Treatment with TMI-1 reduced the secretion of soluble TNF-α and expression of TRPV1 in DRG neurons. Furthermore, the upregulation of TRPV1 induced by paclitaxel increased calcium influx, resulting in the activation of neuroinflammatory signaling proteins (PKC, Akt, PI3K, NF-κB, and phospho-MAPK), but TMI-1 reversed these effects. Therefore, these results demonstrate that TMI-1 counteracts the neurotoxic effect of paclitaxel by contributing to neuroinflammation, which is one of the mechanisms of PIPN.

Paclitaxel induces peripheral neuropathy through multiple mechanisms: 1) arrest of the cell cycle by stabilizing the microtubules, 2) mitochondrial dysfunction, 3) sensitization of ion channels, including voltage-gated sodium channels and TRPV1, and 4) neuronal damage and neuroinflammatory processes (Flatters and Bennett, 2006; Nieto et al., 2008; Kavallaris, 2010; Sisignano et al., 2014; Kamata et al., 2020). The hallmark of PIPN is the degeneration of long axons required for transmitting sensory information. A previous study demonstrated that paclitaxel could lead to axonal blebbing and eventual degeneration, but not neuronal death (Melli et al., 2006). Furthermore, other studies have shown that axons are more vulnerable to paclitaxel-induced neurotoxicity than neuronal cell bodies due to a compartmentalized microfluidic culture platform (Yang et al., 2009). In addition, mouse models of paclitaxel have shown that axonal degeneration occurs in the central and peripheral axon branches of DRG cells (Tasnim et al., 2016). Axonal blebbing and degeneration have been reported in other studies of 50B11 cells treated with cisplatin (Cetinkaya-Fisgin et al., 2020). Our study demonstrated occurrence of axonal blebbing and decreased neurite length in DRG sensory cells after paclitaxel treatment. In contrast, when low-dose paclitaxel was administered to rats through the peritoneum, no other changes were observed, except for endoneurial edema (Polomano et al., 2001).

It has been well known that upregulation of TRPV1 expression in sensory neurons is a hallmark of paclitaxel-induced neurotoxicity (Hara et al., 2013; Li et al., 2015a; Kalynovska et al., 2017). Paclitaxel activates toll-like receptor 4 (TLR4) and sends signals downstream to it to sensitize TRPV1 (Li et al., 2015a). Paclitaxel treatment engages MAPK signaling in NF-κB *via* TLR4 (Li et al., 2015b). Previous research has reported that TNF-α increases TRPV1 expression *via* activation of TLR4 in a paclitaxel-induced neuropathic pain model (Wu et al., 2015). Additionally, TNF-α not only increases intracellular calcium influx but also leads to membrane trafficking of TRPV1 (Meng et al., 2016). In the present study, treatment with paclitaxel increased TNF-α and TRPV1 in 50B11 cells, suggesting that TNF-α induced the overexpression of TRPV1.

Proinflammatory cytokines, such as TNF-α, IL-1β, and IL-6, play a crucial role in the nociceptive process of PIPN (Sisignano et al., 2014). Paclitaxel increases TNF-α production by upregulating TNF-α gene expression in macrophages and endothelial cells (Bogdan and Ding, 1992; Wood et al., 2010). We chose 50B11, an immortalized DRG neuronal line, because these cell lines are suitable for this *in vitro* study since they grow easily and have the characteristics of unmyelinated sensory neurons (Haberberger et al., 2020). The 50B11 cells expressed variable neuronal markers and nociceptors, including markers for C-fiber neurons and TRPV1 in the media containing forskolin. However, glial marker expression has not been observed (Bhattacherjee et al., 2014). In this study, paclitaxel increased the secretion of TNF-α in 50B11 cells. It seemed that the overexpression of TNF-α could be induced by paclitaxel without activation of satellite glial cells.

The secreted TNF-α binds to TNFR, activating ERK 1/2 signaling (Durham, 2006; Utreras et al., 2009; Lei et al., 2012). Several studies have reported that IL-1β and IL-6 regulate the activation of TRPV1 (Rossi et al., 2012; Fang et al., 2015). These cytokines activate protein kinases and phosphorylate TRPV1 (Ma and Quirion, 2007). Intracellular calcium influx through TRPV1 induces the activation of multiple protein kinases, such as PKA and PKC, phosphorylation of MAPK, and activation of NF-κB. Consequently, TRPV1-mediated calcium influx increases proinflammatory cytokines, including IL-1β, IL-6, and TNF-α (Gouin et al., 2017; Kong et al., 2017). The data in the present study indicate that paclitaxel initiates the neuroinflammatory signaling pathway.

The current study also reported that the administration of TMI-1 downregulates the expression of TRPV1. This is considered to be due to TMI-1 administration, which inhibits ADAM17-mediated processing of TNF-α. TMI-1 is a dual inhibitor of ADAM17 and several matrix metalloproteinases (MMPs) (MMP-1, 2, 7, 9, 13, and 14). TMI-1 inhibits the enzymatic activity of ADAM17 and MMPs by binding zinc at the catalytic site (Mezil et al., 2012). A previous study reported that TMI-1 induced apoptosis in tumor cells *via* cell cycle arrest, following the inhibition of MMPs (Mezil et al., 2012). TMI-1 was first developed for the treatment of rheumatoid arthritis. It has been reported that TMI-1 suppresses LPS-induced TNF-α production and reduces the symptomatic clinical severity scores in mouse models of collagen-induced arthritis (Zhang et al., 2004). However, there are differences in the results for IL-1β and IL-6 between our data and theirs. The previous study reported that TMI-1 selectively inhibited TNF-α and did not affect the production of other proinflammatory cytokines, including IL-1β and IL-6, in human monocytes and whole blood. In contrast, the present study reported a decrease in the mRNA levels of IL-1β and IL-6 in differentiated 50B11 DRG cells. This might be because the downregulation of TRPV1 led to the reduction of proinflammatory cytokines.

There are several limitations to the present study. First, this study was limited to an *in vitro* environment with immortalized cells, and it is not clear whether TMI-1 has the same neuroprotective effect *in vivo*. Thus, further *in vivo* clinical studies, such as on rodent pain models of PIPN, should be performed to evaluate the protective effect of TMI-1. Second, our results were attributed to short-term treatment with paclitaxel and TMI-1. A longer duration of treatment may be suitable for the CIPN model because CIPN is a chronic and progressive disease. Third, there seem to be no studies on TMI-1 associated with CIPN. Although this study suggests the possibility of TMI-1 as a therapeutic target for PIPN *via* attenuation of TRPV1 overexpression, further studies on the mechanisms underlying the neuroprotective effect of TMI-1 and on the clinical association between TMI-1 and CIPN are needed. Previous studies have reported that TMI-1 is effective and beneficial in treating arthritis and neoplasm *via* inhibition of ADAM17/MMPs. Considering the cytotoxicity on tumor cells, TMI-1 may be used as an ideal therapeutic agent for both chemotherapy and CIPN.

In conclusion, TMI-1 has a neuroprotective effect on differentiated DRG cells treated with paclitaxel. It reduced the secretion of soluble TNF-α and subsequently decreased the expression of TRPV1. These results suggest that the inhibition of ADAM17 may be beneficial for the treatment of paclitaxel-induced peripheral neuropathy.

## Data Availability

The original contributions presented in the study are included in the article/Supplementary Material, further inquiries can be directed to the corresponding authors.
